# Construction and Interpretation of Prediction Model of Teicoplanin Trough Concentration *via* Machine Learning

**DOI:** 10.3389/fmed.2022.808969

**Published:** 2022-03-08

**Authors:** Pan Ma, Ruixiang Liu, Wenrui Gu, Qing Dai, Yu Gan, Jing Cen, Shenglan Shang, Fang Liu, Yongchuan Chen

**Affiliations:** ^1^Department of Pharmacy, The First Affiliated Hospital of Third Military Medical University (Army Medical University), Chongqing, China; ^2^Department of Clinical Pharmacy, General Hospital of Central Theater Command of PLA, Wuhan, China

**Keywords:** machine learning, SHAP, precision medicine, prediction model, model explanation, algorithm, teicoplanin

## Abstract

**Objective:**

To establish an optimal model to predict the teicoplanin trough concentrations by machine learning, and explain the feature importance in the prediction model using the SHapley Additive exPlanation (SHAP) method.

**Methods:**

A retrospective study was performed on 279 therapeutic drug monitoring (TDM) measurements obtained from 192 patients who were treated with teicoplanin intravenously at the First Affiliated Hospital of Army Medical University from November 2017 to July 2021. This study included 27 variables, and the teicoplanin trough concentrations were considered as the target variable. The whole dataset was divided into a training group and testing group at the ratio of 8:2, and predictive performance was compared among six different algorithms. Algorithms with higher model performance (top 3) were selected to establish the ensemble prediction model and SHAP was employed to interpret the model.

**Results:**

Three algorithms (SVR, GBRT, and RF) with high *R*^2^ scores (0.676, 0.670, and 0.656, respectively) were selected to construct the ensemble model at the ratio of 6:3:1. The model with *R*^2^ = 0.720, MAE = 3.628, MSE = 22.571, absolute accuracy of 83.93%, and relative accuracy of 60.71% was obtained, which performed better in model fitting and had better prediction accuracy than any single algorithm. The feature importance and direction of each variable were visually demonstrated by SHAP values, in which teicoplanin administration and renal function were the most important factors.

**Conclusion:**

We firstly adopted a machine learning approach to predict the teicoplanin trough concentration, and interpreted the prediction model by the SHAP method, which is of great significance and value for the clinical medication guidance.

## Introduction

Teicoplanin is a glycopeptide antibiotic for the treatment of severe infections caused by Gram-positive bacteria, including methicillin-resistant *Staphylococcus aureus* (MRSA) (1). As an alternative to vancomycin, teicoplanin shows comparable clinical outcomes with fewer occurrences of nephrotoxicity, ototoxicity, and red man syndrome (2). However, with a very highly bound to plasma albumin, teicoplanin has a very long terminal elimination half-life (ranging from 100 to 170 h) and even takes several days to achieve the effective plasma concentration, which results in a great individual variability and permitting once daily dose (3). Therefore, an initial loading dose is required to achieve effective plasma concentration rapidly (3). Teicoplanin is highly bioavailable (>90%) and eventually excreted in urine as a prototype. Because of these pharmacokinetic characteristics, the fixed dosing regimens of teicoplanin administered to patients suffering from hypoalbuminemia (3), and/or renal insufficiency, and/or an expansion of the extracellular fluids might lead to the wide variations and fluctuations of concentrations (4).

The plasma trough concentration of teicoplanin is closely associated with its therapeutic efficacy. A large number of studies have shown that treated with the conventional regimen, many patients may fail to reach therapeutic targets that lead to clinical failure. However, repeated exposure to suboptimal concentrations increases the risk factor of teicoplanin resistance (5, 6). According to previous studies, 10–30 mg/l was regarded as the target teicoplanin trough level for successful treatment (5, 6). The teicoplanin trough concentrations are mainly influenced by the teicoplanin administration regimen and the patient's pathophysiological conditions (such as age, weight, serum albumin, renal function, concomitant therapy, concomitant diseases, etc.) (4).

Customization of the antimicrobial dosing regimen is continuously gaining more relevance in the antimicrobial stewardship programs (7, 8). In this regard, therapeutic drug monitoring (TDM), by measuring drug exposure in plasma, may be helpful in individual therapies (3). TDM is an effective method that assures adequate trough concentration for maximum efficacy and thus, prevents adverse effects resulting from overexposure (8–10). Based on the daily monitoring of teicoplanin concentration on our TDM platform, individual variation is evident, with low concentrations of teicoplanin, most of which are unable to reach an effective plasma trough concentration. However, some hospitals have no TDM platform due to the limited medical conditions, and sampling and testing of TDM cost time and money. In order to bring convenience to clinicians and save time and money for patients, more than TDM, more powerful drug concentration prediction tools are needed.

Machine learning algorithms, as a subdiscipline of artificial intelligence, take advantage of large-scale complex algorithms and datasets to uncover useful patterns, that can evaluate data-driven estimation when forecasting from multiple variables and obtain nonlinear variable relations to deliver predicted clinical outcomes with high accuracy (11, 12). The rapidly developing machine learning has been widely applied in the biomedicine field, such as clinical diagnostics, precision treatments, and health monitoring (13). However, population pharmacokinetic (PPK) models are adopted by the ongoing research on teicoplanin trough concentration. It includes certain criteria such as age, weight, and creatinine/creatinine clearance rate (8, 14). Few studies on the prediction of teicoplanin trough concentration have adopted machine learning to model. In this study, the machine learning approach was employed to establish an optimal ensemble model to predict the teicoplanin trough concentrations, which can assist clinicians in guiding the dosage of medication. Furthermore, the SHapley Additive exPlanation (SHAP) method was used to explain the feature importance in our ensemble prediction model, so that our study could also provide a reasonable explanation for the prediction, which demonstrated how the relevant factors influenced the teicoplanin trough concentrations.

## Methods

### Patients and Data

A retrospective study was conducted among patients who underwent teicoplanin intravenously at the First Affiliated Hospital of Army Medical University from November 2017 to July 2021. Patients were enrolled in this study according to the following inclusion criteria: (1) age > 14 years; (2) > 2–3 days of treatment with teicoplanin (steady-state concentration); and (3) underwent TDM of teicoplanin in which the trough blood samples were collected immediately before administering the next dose. The following exclusion criteria were applied: (1) pregnant women and (2) failed to reach the lower limit of quantification (LLOQ) for teicoplanin through concentration assay.

### Ethics Approval

This study was approved by the Hospital Ethics Committee of the Southwest Hospital of Army Medical University ([B]KY2021095) and performed in accordance with the Declaration of Helsinki. In the ethical approval documents, the informed consent has been exempted. The procedures in this study are fully compliant with the ethical standards in accordance with the Institutional Research Committees.

### Measurement of Teicoplanin Trough Concentration

The teicoplanin plasma trough concentration was measured by high-performance liquid chromatography (HPLC) (1200 Series, Agilent Technologies Incorporation). Determination was performed using the Innoval-C_18_ column (5 μm, 4.6 mm × 250 mm, Dikma Technologies). The mobile phase was 76% sodium dihydrogen phosphate (0.01 mmol/L) and 24% acetonitrile (pH 2.9). The UV detection wavelength was 240 nm. The trough plasma concentration linear range was 3.125–100.000 mg/l (correlation coefficient *R*^2^ = 0.9998). Both the intra- and interday precisions were within 7%.

### Data Collection and Processing

The teicoplanin dataset includes teicoplanin administration (loading dose, time of loading dose, loading intervals, maintenance intervals, and total duration of treatment), demographic information (age, height, weight, gender, and APACHE II), laboratory parameters [albumin (ALB), estimated glomerular filtration rate (eGFR), cystatin C (Cys-C), creatinine clearance rate (CLcr), aspartate aminotransferase (AST), alanine aminotransferase (ALT), TBIL, NEU%, and PLT], concomitant therapy (ECMO, CRRT, and co-medication), and concomitant diseases (AML, hyperproteinemia, sepsis) were obtained from the hospital's electronic medical record system (EMRS). After cleaning up of teicoplanin dataset, the target variable and relevant crucial covariates were screened subsequently. The rate of missing data is 3.32%. The mean filling method in Python (version 3.6, Python Software Foundation) was employed to fill the missing data, resulting in a dataset of 279 × 27. The teicoplanin trough concentrations were selected as the target variable, while the whole dataset was randomly divided into a training group and testing group at the ratio of 8:2.

### Modeling and Validation

The linear correlation between the teicoplanin trough concentrations and the relevant covariates was evaluated (Supplementary Table S1). According to the correlation coefficient, the linear correlation among them was poor. Therefore, six nonlinear machine learning algorithms for modeling were employed to predict the teicoplanin trough concentrations, including support vector regression (SVR), random forest (RF), Adaptive Boosting (Adaboost), Boostrap aggregating (Bagging), Gradient Boosted Regression Trees (GBRT), and eXtreme Gradient BoostingX (XGBoost).

In order to evaluate the single algorithm predictive performance, the metrics of *R*-squared (*R*^2^), mean square error (MSE), and mean absolute error (MAE) were used. *R*^2^ indicates the explanation degree of the independent variable to the dependent variable. The proportion of a single algorithm in the final model was determined through the prediction of different algorithms. The final result of the ensemble model is the weighted average based on the ranking of the top three algorithms. The calculating formulas are as follows:


$$\begin{array}{l} MAE\left(y^{o},y^{p}\right)=\frac{1}{N}\sum_{i=1}^{N}|y_{i}^{o}-y_{i}^{p}|\\ MSE\left(y^{o},y^{p}\right)=\frac{1}{N}{\sum_{i=1}^{N}\left(y_{i}^{o}-y_{i}^{p}\right)}^{2}\\ R^{2}\left(y^{o},y^{p}\right)=1-\frac{{\sum_{i=1}^{N}\left(y_{i}^{o}-y_{i}^{p}\right)}^{2}}{\sum_{i=1}^{N}\left(y_{i}^{o}-\bar{y^{o}}\right)}\\ \bar{y^{o}}=\frac{1}{N}\sum_{i=1}^{N}y_{i}^{o} \end{array}$$

*R*^2^ represents the goodness of fit of the model, and the value range is 0–1. The closer *R*^2^ gets to 1, the better the goodness of fit of the model becomes.*y*^*o*^ represents the observed value; *y*^*p*^ represents the predicted value. With reference to MSE and MAE, when their values decrease, the model has improved the goodness of fit. In addition, the accuracy of predicted trough concentration compared with the observed concentration was investigated. The absolute accuracy represented the accuracy of the predicted trough concentration to be within ± 5 mg/L of the observed trough concentration, while the relative accuracy showed that the predicted trough concentration was within ± 30% of the observed trough concentration.

**Figure 1:**
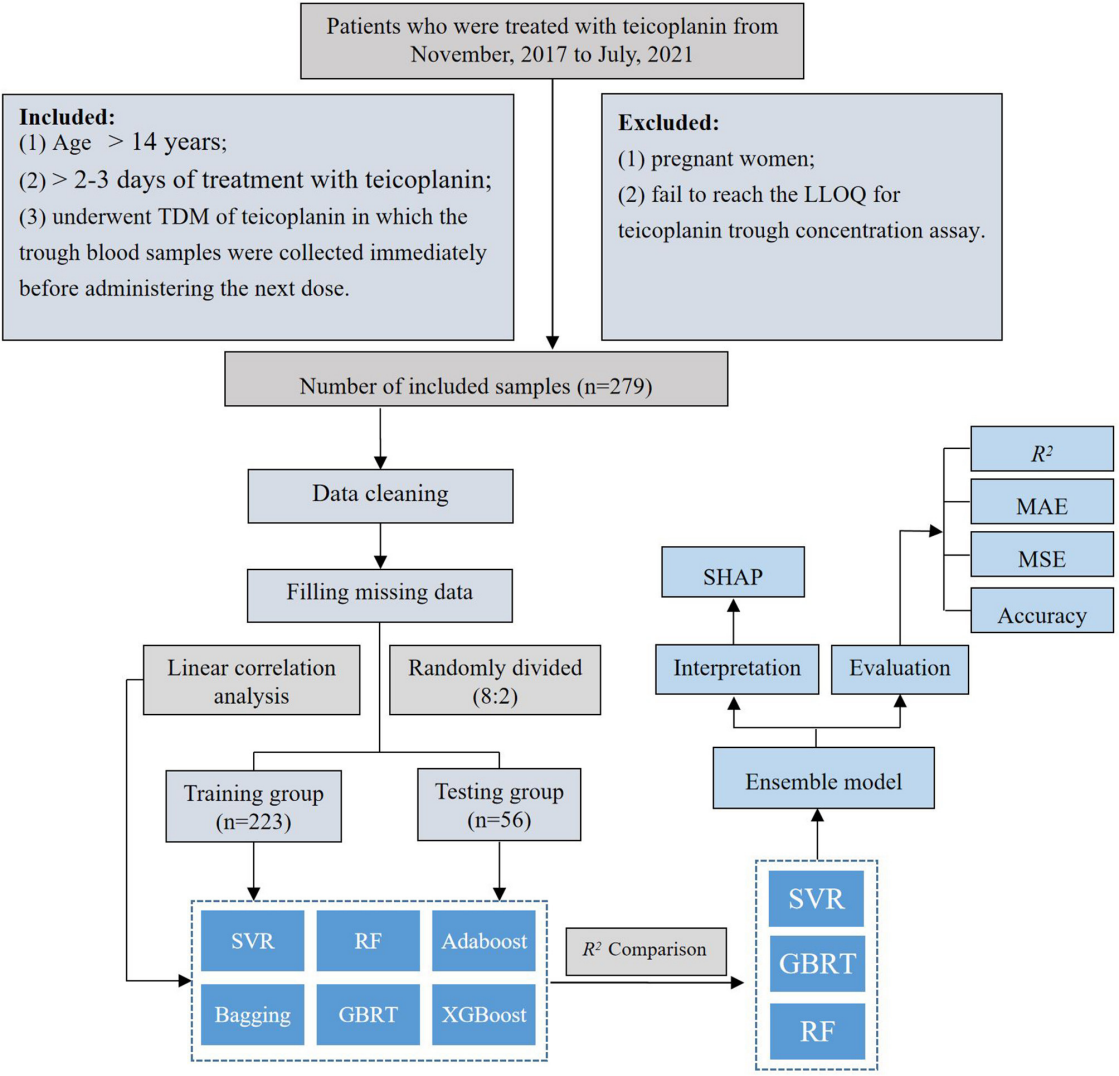
The workflow of data processing and algorithm selection.

The top three algorithms were selected to establish the ensemble prediction model of teicoplanin trough concentrations. In addition, another dataset of 20 patients were collected as the validation group to corroborate the performance of the prediction models. The workflow of data processing, algorithm selection, and modeling were displayed in Figure 1.

### Model Interpretation

SHapley Additive exPlanation, is a game-theoretic method that provides information to machine learning outputs. It determines and allocates credit for model outputs by means of Shapley values coming from game theory including all related covariants (15). As an additive feature attribution method, SHAP value represents contributions of each feature in a certain sample, in which each feature is regarded as a “contributor.” A feature with a positive SHAP value improves the output value, and those larger numerical values make greater contributions (16, 17). SHAP values were used to provide the interpretation of our ensemble prediction model (18), in which the SHAP summary plot, the importance ranking, and the SHAP dependence plot of the relevant covariates were demonstrated based on the permutation explainer provided by the SHAP Python package (version 0.39.0).

### Statistical Analysis

Statistical analysis was performed using IBM SPSS version 25.0 (IBM Corporation, Armonk, New York, USA). The Kolmogorov–Smirnov test was used to evaluate whether the measurement data were normally distributed. Measurement data were presented as the median and interquartile range (IQR) for nonnormal distribution variables and mean ± SD for normal distribution variables. Measurement data were analyzed by Mann-Whitney *U* test (non-normal distribution) and independent *t*-test (normal distribution). Categorical data were expressed as *n* (%) and analyzed by the chi-squared test (*n* ≥ 5) or Fisher's exact test (*n* < 5). The tests were two-sided with a *p* < 0.05 which deemed statistically significant.

## Results

### Baseline Patient Characteristics

This study was performed on 279 TDM measurements obtained from 192 patients who underwent teicoplanin treatment. The whole dataset was randomly divided into training group and testing group at the ratio of 8:2, which were 223 and 56 cases, respectively. The baseline information of 27 variables and the comparison between the training and testing groups were shown in Table 1, without any significant difference between variables of the two groups (*p* > 0.05).

**Table 1:** The description of the study samples.

**Variables**	**Values**		***P* value**
	**Training (*n* = 223)**	**Testing (*n* = 56)**	
Teicoplanin trough concentration (mg/L)	13.32 (8.87,19.12)	11.291 (7.62,22.11)	0.603 ^a^
Teicoplanin administration			
Loading dose (mg/kg)	7.91 (6.67, 12.18)	7.45 (6.09, 12.23)	0.149 ^a^
Times of loading dose			0.747 ^d^
<3	68 (30.49)	14 (25)	
3-5	149 (66.82)	41 (73.21)	
> 5	6 (2.69)	1 (1.79)	
Loading intervals (h)			0.924 ^d^
12	201 (90.13)	52 (92.86)	
24	14 (6.28)	3 (5.36)	
Others	8 (3.59)	1 (1.79)	
Maintenance dose (mg/kg)	8 (6.09, 12.18)	7.08 (5.64, 12.23)	0.086 ^a^
Maintenance intervals (h)			0.249 ^d^
12	13 (5.83)	2 (3.57)	
24	196 (87.89)	47 (83.93)	
Others	14 (6.28)	7 (12.5)	
Total duration of treatment (day)	5 (3, 8)	4 (3, 6.75)	0.079 ^a^
Demographic information			
Age (years)	53 (40, 66)	52.5 (38.25, 65.75)	0.823 ^a^
Height (cm)	165 (155.07, 168.27)	165 (155.07, 170)	0.782 ^a^
Weight (kg)	59 (53.44, 65.7)	65 (53.44, 73.75)	0.059 ^a^
Gender, male (n, %)	80 (35.87%)	25 (44.64%)	0.226 ^c^
APACHE II	24(20, 28)	23(19, 26)	0.053^a^
Laboratory parameters			
ALB (g/L)	32.32 ± 4.91	32.40 ± 4.59	0.911 ^b^
eGFR (ml/min/L)	97.06 (61.84, 120.57)	86.63 (50.61, 111.41)	0.063 ^a^
Cys-C (mg/L)	1.52 (0.97, 1.7)	1.6 (1.05, 1.76)	0.453 ^a^
CLcr ^e^ (mL/min/1.73m^2^)	85.55 (55.43, 125.25)	78.23 (40.22, 123.97)	0.317 ^a^
AST (IU/L)	41.7 (22.5, 79.9)	34.2 (19.63, 81.6)	0.754 ^a^
ALT (IU/L)	26.5 (14, 49.2)	24.8 (10.78, 69.25)	0.695 ^a^
TBIL (umol/L)	20.4 (12.4, 48.1)	19.25 (13.93, 50.05)	0.984 ^a^
NEU%	77.1 (64.5,87.5)	79.5 (67.53,89.1)	0.457 ^a^
PLT (10^9^/L)	83 (37, 196)	135.5 (40, 252.25)	0.142 ^a^
Concomitant therapy			
ECMO (*n*, %)	11 (4.93%)	2 (3.57%)	1.000 ^d^
CRRT (*n*, %)	61 (27.35%)	16 (28.57%)	0.855 ^c^
Co-medication ^f^ (*n*, %)	17 (7.62%)	3 (5.36%)	0.774 ^d^
Concomitant diseases			
AML (*n*, %)	30 (13.45%)	8 (14.29%)	0.871 ^c^
Hypoproteinemia (*n*, %)	143 (64.13%)	34 (60.71%)	0.636 ^c^
Sepsis, (*n*, %)	80 (35.87%)	14 (25%)	0.110 ^c^

*APACHE II, acute physiology and chronic health evaluation II; ALB, albumin; Cys-C, cystatin C; eGFR, estimated glomerular clearance; CLcr, creatinine clearance rate; AST, aspartate aminotransferase; ALT, alanine aminotransferase; TBIL, total bilirubin; NEU%, the percentage of neutrophils; PLT, platelet count; ECMO, extracorporeal membrane oxygenation; CRRT, continuous renal replacement therapy, AML, acute myeloid leukemia*.

*Measurement data were presented as median and interquartile range (IQR) for non-normal distribution variables and mean ± SD for normal distribution variables. Categorical data were expressed as n (%)*.

a *Mann–Whitney U test*.

b *Independent t-test*.

c *Chi-squared test*.

d *Fisher's exact test*.

e *Creatinine clearance was calculated by the Cockcroft formula. CLcr = (140 – age [years]) × weight (WT, kg) × 0.85 (if female)/0.818 × SCr (μmol/L)*.

f *Comedication included Furosemide, Amikacin Sulfate, Cyclosporine, Isepamicin Sulfate, Amphotericin B liposome and Colistin Sulfate*.

### Algorithm Selection

According to the linear correlation result (Supplementary Table S1), the linear correlation between the teicoplanin trough concentrations and the relevant covariates was poor. Thus, six nonlinear algorithms were included for the algorithm selection. The performance metrics of six different algorithms including *R*^2^, MAE, MSE, and accuracy were shown in Table 2. Among the six algorithms, SVR has the best predictive performance of prediction, with the highest *R*^2^, accuracy, and lowest MAE, MSE. To select the algorithms to establish the ensemble prediction model for further promoting stability and accuracy, *R*^2^ was chosen to evaluate the goodness-of-fit of the model. Among the six algorithms, SVR, GBRT, and RF had high goodness-of-fit, which is 0.676, 0.670, and 0.656, respectively. As a result, the top three performing algorithms (SVR, GBRT, and RF) were chosen to predict teicoplanin trough concentration and for a subsequent experiment.

**Table 2:** The model performance metrics of six different algorithms.

**Model**	***R^**2**^***	**MAE**	**MSE**	**Accuracy-1 ^**a**^**	**Accuracy-2 ^**b**^**
SVR	0.676	3.868	26.071	76.79%	62.50%
GBRT	0.670	4.054	26.568	69.64%	62.50%
RF	0.656	4.410	27.683	62.50%	48.21%
Bagging	0.652	4.440	28.059	64.29%	44.64%
Adaboost	0.610	4.743	31.386	55.36%	48.21%
XGBoost	0.551	4.630	36.186	60.71%	55.36%

*SVR, Support Vector Regression; GBRT, Gradient Boosted Regression Trees; RF, Random Forest; Bagging, Boostrap aggregating; Adaboost, Adaptive Boosting; XGBoost, eXtreme Gradient Boosting*.

a *Absolute accuracy, the predict trough concentration was within ± 5 mg/l of the observed trough concentration*.

b *Relative accuracy, the predict trough concentration was within ± 30% of the observed trough concentration*.

### Modeling and Validation

**Figure 2:**
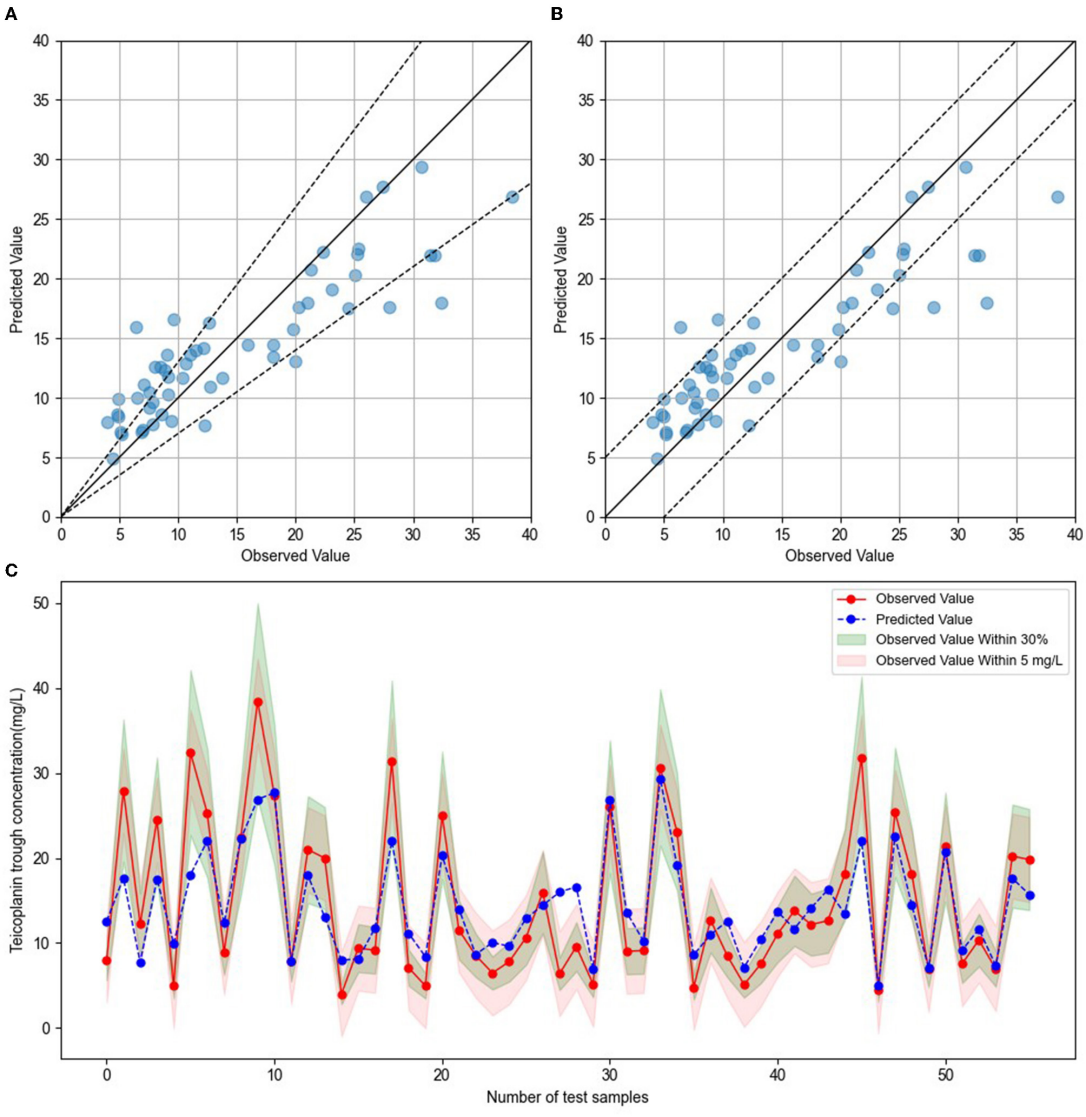
Comparison of predicted and observed value. **(A)** The blue dots represented testing sample, with observed values on the x-axis and predicted values on the y-axis. The blue dots between the dotted lines indicated that the predict values were within ± 30% of the observed values. **(B)** The blue dots represented testing sample, with observed values on the x-axis and predicted values on the y-axis. The blue dots between the dotted lines indicated that the predict values were within ± 5 mg/l of the observed values. **(C)** The red dots indicated the observed values, and blue dots indicated the predicted values. The green shade represented within ± 30% of the observed values, and the red shade represented within ± 5 mg/l of the observed values.

To establish the ensemble prediction model of teicoplanin trough concentration, the target parameters were set as the highest *R*^2^, absolute accuracy, and relative accuracy, then the weight proportion of three candidate algorithms (SVR, GBRT, and RF) with a high *R*^2^ score was adjusted. Based on the automatic calculations of machine learning, the ensemble model composed of SVR, GBRT, and RF (6:3:1) was determined. Compared to any single algorithm, the ensemble model had the best performance with the highest *R*^2^, absolute accuracy and lowest MAE, MSE (Table 3). Based on the testing group's data, the absolute accuracy (± 5 mg/l) of the ensemble model was 83.93%, and the relative accuracy (± 30%) was 60.71%. To validate the ensemble model, another dataset of 20 patients were collected from the hospital as the validation group. The results showed that validation group had higher relative accuracy and lower MAE, MSE than the testing group (Table 3), indicating that the model has quite good generalization ability. The exact distribution of predicted and observed values for teicoplanin trough concentration was shown in Figure 2.

**Table 3:** The model performance metrics of the ensemble model.

**Group**	***R^**2**^***	**MAE**	**MSE**	**Accuracy-1 ^**a**^**	**Accuracy-2 ^**b**^**
Testing group	0.720	3.628	22.571	83.93%	60.71%
Validation group	0.686	3.196	18.260	77.78%	88.89%

a * Absolute accuracy, the predict trough concentration was within ± 5 mg/l of the observed trough concentration*.

b *Relative accuracy, the predict trough concentration was within ± 30% of the observed trough concentration*.

### Interpretation of the Ensemble Model

**Figure 3:**
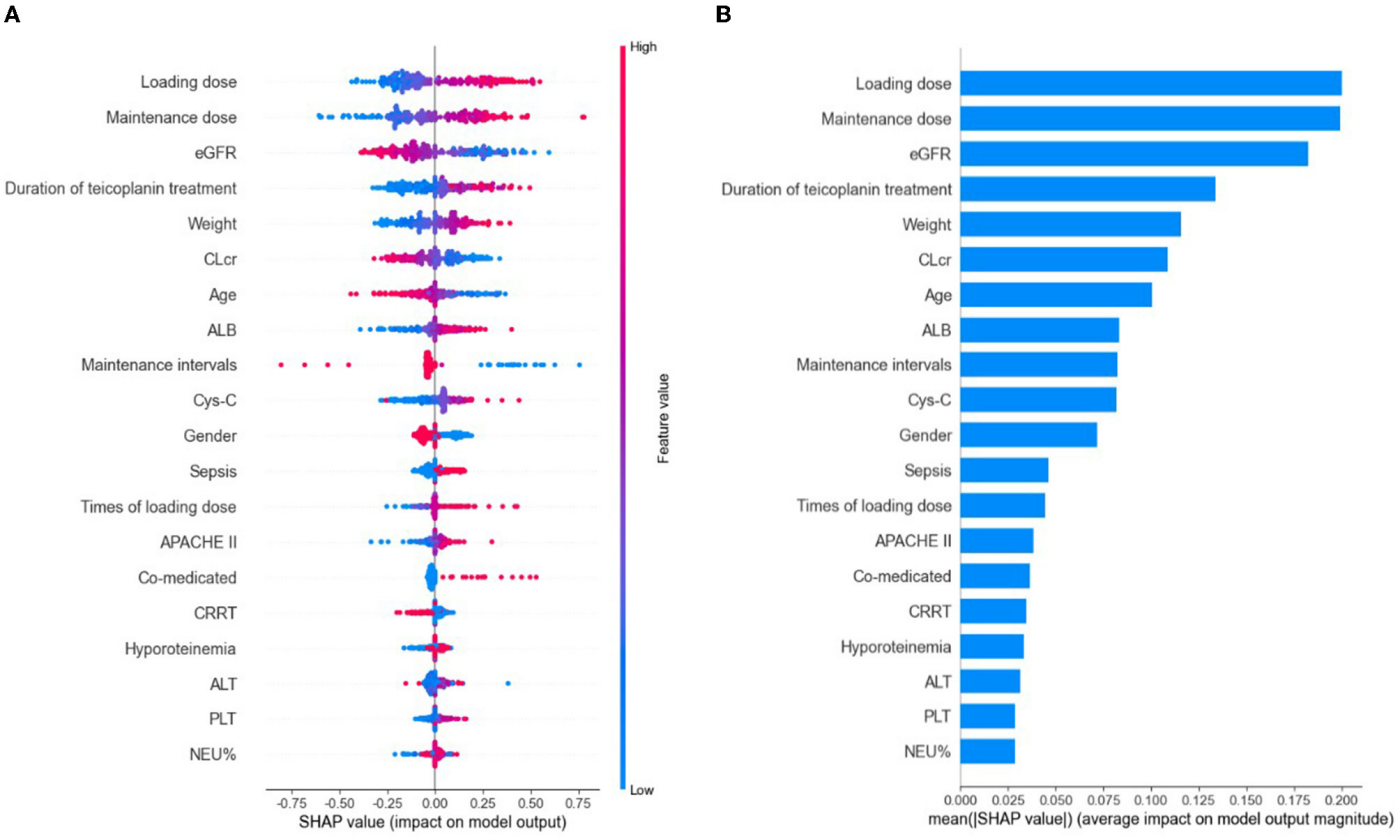
The model's interpretation by SHapley Additive exPlanation (SHAP). eGFR, estimated glomerular clearance; CLcr, creatinine clearance rate; ALB, albumin; Cys-C, cystatin C; APACHE II, Acute Physiology and Chronic Health Evaluation II; CRRT, continuous renal replacement therapy; ALT, alanine aminotransferase; PLT, platelet count; NEU%, the percentage of neutrophils. **(A)** The SHAP summary plot of the top 20 relevant variables. The SHAP value (x-axis) is a unified index responding to the effect of a variable in the ensemble model. In each variable importance row, all the patients' attributes to the outcome were plotted using different colored dots, in which the red (blue) dots represent high (low) values. The higher the SHAP value of a variable, the higher teicoplanin trough concentration. **(B)** The importance ranking of the top 20 variables according to the mean (|SHAP value|).

Based on the selected relevant variables, the SHAP figures demonstrated the positive or negative correlations between the relevant variables and the teicoplanin trough concentrations. The SHAP summary plot of the top 20 relevant variables in the ensemble model was displayed in Figure 3A. The feature values ranked the importance of the prediction model, with loading dose and maintenance dose on the top two. The dot color represents the feature values of each variable, which is redder when the feature value gets higher and bluer when the feature value gets lower. Each feature value of a certain variable corresponds to a SHAP value (x-axis). For one sample, the aggregation of the SHAP values of each variable equals to the predicted teicoplanin trough concentration. To identify the features that influenced the ensemble model the most, the average of absolute SHAP values of each relevant variable (top 20) was calculated, the top 12 of which included loading dose, maintenance dose, eGFR, duration of teicoplanin treatment, weight, CLcr, age, ALB, maintenance intervals, Cys-C, gender, and sepsis in a descending order. Among them, the SHAP value of loading dose has the highest score (0.200), followed by the SHAP value of maintenance dose (0.199), and eGFR (0.182) demonstrating their importance in predicting the teicoplanin trough concentration (Figure 3B).

**Figure 4:**
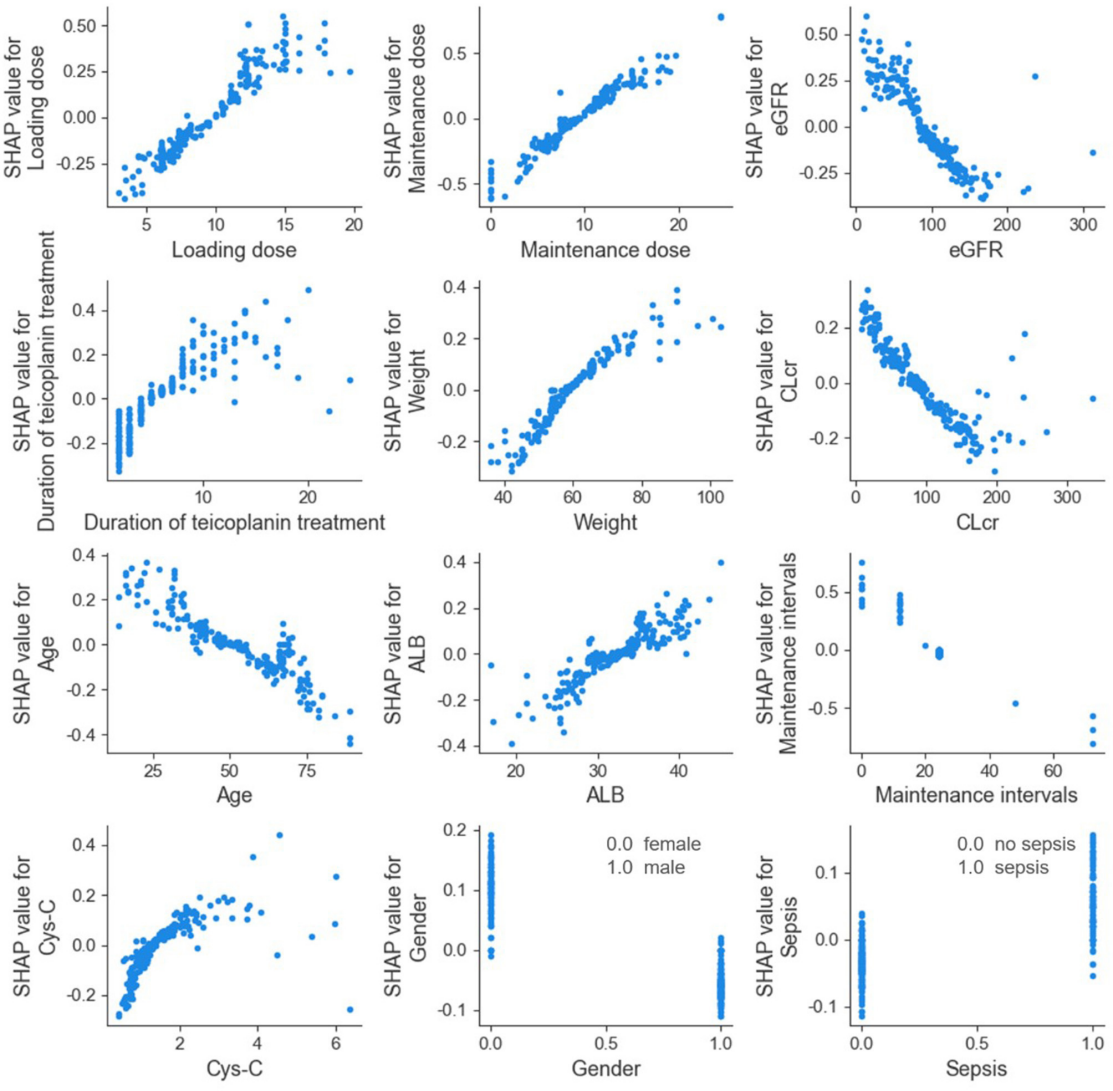
SHAP dependence plot of model. eGFR, estimated glomerular filtration rate; CLcr, creatinine clearance rate; ALB, albumin; Cys-C, cystatin C. The SHAP dependence plot showed how the relevant variable affected the output of the ensemble prediction model. SHAP values for specific relevant variable exceed 0, representing an increased teicoplanin trough concentration.

The SHAP dependence plot of the top 12 relevant variables was displayed in Figure 4. Our results showed higher loading dose, maintenance dose, duration of teicoplanin treatment, weight, ALB, Cys-C, as well as lower eGFR, CLcr and age were related to higher teicoplanin trough concentration. Female patients and patients with sepsis comorbidities may have higher teicoplanin trough concentration.

## Discussion

Herein, we constructed an optimal prediction model of teicoplanin trough concentration, and used SHAP method to interpret of the prediction model. We selected the algorithms through *R*^2^ comparison and continuously debug the ratio to optimize the ensemble model. Ultimately, SVR, GBRT, and RF (6:3:1) were determined, of which the *R*^2^ and the absolute accuracy exceeded any single algorithm, and the MAE, MSE were lower than any single algorithm. The SHAP values demonstrated the feature importance and direction of each variable, and clarified the correlation between the target variable and the relevant important covariates, which is of great significance and value for the clinical medication guidance.

Machine learning is used broadly in the biomedicine field. Its main ability is to gather and interpret any relevant data even on a large scale and thus, transforms medicine to a data-driven approach. Precision treatment is one of the top applications of machine learning, where a patient receives tailored medical care, such as personalized dose adjustment, plasma concentration prediction, and adverse drug events prediction (19–22). Ensemble learning, one of the key features of machine learning, comes from a combination of various models that is capable of producing a final prediction. Random forests, gradient boosting, and stacking/meta-ensembles are some of the approaches available in this feature (13). In this study, the ensemble model performed better than any single algorithm included by contrasting the goodness-of-fit and accuracy.

The traditional pharmacokinetic analysis is based on mathematically simple techniques with poor applicability and high requirements for data quality (23). PPK analysis, a new statistical approach, combines the traditional pharmacokinetic model with population statistics model, of which nonlinear mixed-effects modeling (NONMEM) is the most widely used program (23). However, owing to the explicit analytical model used, PPK model is relatively rigid to apply, where adding or removing a parameter may be complicated (24). In contrast, self-organization is what makes up machine learning. It enables computers to access previous data without being explicitly programmed. Many researches have reported that the predicting accuracy of machine learning approach exceeded the PPK method (20, 25). Huang et al. constructed an ensemble prediction model of vancomycin trough concentrations, and compared with PPK model. Their findings showed that machine learning model works better with higher accuracy of prediction (20). The evaluation parameters (*R*^2^ and accuracy) of our ensemble predicting model have surpassed its vancomycin counterpart, suggesting that our model has a good prediction effect and prospect of clinical application.

The interpretation of predictions from a complex statistic model might make equal sense to the model prediction itself in healthcare (26). As a classic *posthoc* interpretation method, SHAP identifies the significant influencing factors with its effect magnitude (27). In this study, the distribution of SHAP values of a relevant covariate, and also its importance and direction were measured. The averages of absolute SHAP values indicated that teicoplanin administration was the most important factor, for which the loading dose, maintenance dose, duration of teicoplanin treatment and maintenance intervals ranked first, second, fourth, and ninth, respectively. Due to its long elimination half-life, teicoplanin requires ample time for the concentration to achieve constant state. As a result, loading doses are required to exhibit the same concentration promptly. It has been reported that increase of loading doses is beneficial for the clinical outcomes, but significant teicoplanin underexposure onset of the therapy is imminent if insufficient dosing persists (28, 29), which were consistent with our study. The SHAP dependence plot showed that the teicoplanin trough concentration was positively correlated with loading dose, maintenance dose, duration of teicoplanin treatment, and negatively correlated with maintenance intervals. It indicated that sufficient loading dose should be ensured first to rapidly achieve the effective plasma concentration, and on this basis, adequate maintenance dose, treatment duration and appropriate maintenance intervals were also necessary.

Since teicoplanin is mainly eliminated as prototype through the kidney, renal dysfunction causes a prolongation of the elimination half-life and an elevated plasma concentration of teicoplanin (28). A large number of studies have demonstrated that renal function-related parameters including eGFR, CLcr, and Cys-C were the significant covariate influencing teicoplanin elimination (9, 14, 30, 31). The concomitant diseases and medication that affect the renal function can also influence teicoplanin trough concentration. For example, sepsis is often accompanied by multiple organ dysfunction, including renal insufficiency, leading to plasma accumulation of teicoplanin due to the reduced elimination. Co-medication with drugs that are explicitly warned by instructions with a high risk of exacerbating renal toxicity, also increases the metabolic burden of renal function and affects the elimination of teicoplanin. Consistent with our findings, our results showed that low level of eGFR and CLcr, as well as high level of Cys-C were closely related to higher teicoplanin trough concentration, with the importance ranking third, sixth, and tenth, respectively. Moreover, patients with sepsis comorbidities and comedication might have higher teicoplanin trough concentration. Furthermore, the level of plasma ALB was another important factor that affects the teicoplanin trough concentration. With a high-binding rate of plasma ALB (90–95%), most teicoplanin combine with plasma ALB as teicoplanin-ALB complex (32). Our results demonstrated that ALB was positively related with the teicoplanin trough concentration, ranking eighth in importance. For patients with hypoalbuminemia, ALB supplementation should be the first priority, which matters not only for the drug treatment, but for maintaining the normal physical function. Meanwhile, shortening the loading interval and appropriately increasing the loading dose can be a feasible measure. Researches have shown that the concomitant therapy such as continuous renal replacement therapy (CRRT) and extracorporeal membrane oxygenation (ECMO) may interfere with the pharmacokinetics of teicoplanin (33), for which drugs may be cleared during *in vitro* CRRT or adhere to the fibers and catheters of oxygenator during ECMO (34, 35). Consistently, our results indicated that the teicoplanin trough concentrations of patients with ECMO and CRRT therapy showed a downward trend. In our study, pediatric patients (aged <14 years) were excluded because of their diverse pharmacokinetics (36). According to the medication instruction, no dose adjustment is required for the elderly patients. However, our SHAP values showed that age was positively related with the teicoplanin trough concentration, which might result from the commonly concomitant therapy for elders. Fan et al. found that gender affected the tigecycline trough plasma concentration in ICU patients, and women were independent risk factors for high-tigecycline exposure (37). Similar results were obtained in our SHAP analysis that female patients have higher teicoplanin trough concentration compared with male. Thus, we suggest to take all the aforementioned factors into account in the teicoplanin administration regimen.

Despite the promising results, there is room to optimize our ensemble prediction model overall. Considerable limitations of this study should be taken into account. First, due to limited samples on hand, accuracy may be compromised. Construction of the model itself calls for a modest number of samples, let alone further modeling that the study may deem necessary. Second, since retrospective data rather than prospective data were used in the study, some uncontrollable factors were inevitable. For instance, the fluctuation in blood collection time point might lead to changes in the teicoplanin plasma concentration. Third, an external validation should be performed in the future studies to improve the applicability of this model.

Our study primarily aims to encourage the application of machine learning methods in biomedicine. To the best of our knowledge, scarcely any study has adopted machine learning approach to predict the teicoplanin trough concentration yet, and we firstly used SHAP values to interpret of the ensemble algorithm model. Therefore, our study fills the gap in this research field. In the future, we plan to further establish an easy-to-use web application based on the presented prediction model, which then could serve as a real-time support tool in clinical decision by self-learning and optimizing, and to help with the personalized dose adjustment of teicoplanin.

## Data Availability Statement

The original contributions presented in the study are included in the article/Supplementary Material, further inquiries can be directed to the corresponding authors.

## Author Contributions

PM: conception and design of the study, acquisition of data, and drafting the article. RL, QD, and YG: acquisition of data. WG and JC: analysis and interpretation of data. SS: drafting the article and analysis and interpretation of data. FL: conception and design of the study and revising it critically for important intellectual content. YC: conception and design of the study and final approval of the version to be submitted. All authors contributed to the article and approved the submitted version.

## Funding

This study was supported by the Talent Pool Program of the Army Medical University (XZ-2019-505-073).

## Conflict of Interest

The authors declare that the research was conducted in the absence of any commercial or financial relationships that could be construed as a potential conflict of interest.

## Publisher's Note

All claims expressed in this article are solely those of the authors and do not necessarily represent those of their affiliated organizations, or those of the publisher, the editors and the reviewers. Any product that may be evaluated in this article, or claim that may be made by its manufacturer, is not guaranteed or endorsed by the publisher.
