# Supplementary material for: Construction and Interpretation of Prediction Model of Teicoplanin Trough Concentration via Machine Learning
Source: Front Med (Lausanne). 2022 Mar 8;9:808969. doi: 10.3389/fmed.2022.808969 (PMC8963816; doi:10.3389/fmed.2022.808969)
Supplement: Supplementary file 1 [file Data_Sheet_1.docx]

**Supplementary Data**

# Table S1. The linear correlation between the teicoplanin trough concentrations and the relevant covariates.

| **Variables** | **Correlation Coefficient ^a^** |
| --- | --- |
| Trough Concentration | 1.000 |
| Loading dose | 0.474 |
| Times of loading dose | 0.168 |
| Loading intervals | -0.058 |
| Maintenance dose | 0.369 |
| Maintenance intervals | -0.015 |
| Duration of teicoplanin treatment | 0.136 |
| Age | -0.052 |
| Height | -0.018 |
| Weight | -0.067 |
| Gender | -0.089 |
| APACHE II | -0.111 |
| ALB | 0.190 |
| eGFR | -0.264 |
| Cys-C | 0.221 |
| CLcr | -0.289 |
| AST | 0.012 |
| ALT | 0.004 |
| TBIL | -0.015 |
| NEU% | 0.096 |
| PLT | 0.003 |
| ECMO | -0.063 |
| CRRT | 0.105 |
| Co-medicated | 0.137 |
| AML | -0.047 |
| Hyporoteinemia | -0.002 |
| Sepsis | 0.229 |

^a^ Pearson correlation coefficient

Abbreviations: APACHE II, acute physiology and chronic health evaluation II; ALB, albumin; Cys-C, cystatin C; eGFR, estimated glomerular clearance; CLcr, creatinine clearance rate; AST, aspartate aminotransferase; ALT, alanine aminotransferase; TBIL, total bilirubin; NEU%, the percentage of neutrophils; PLT, platelet count; ECMO, extracorporeal membrane oxygenation; CRRT, continuous renal replacement therapy, AML, acute myeloid leukemia.


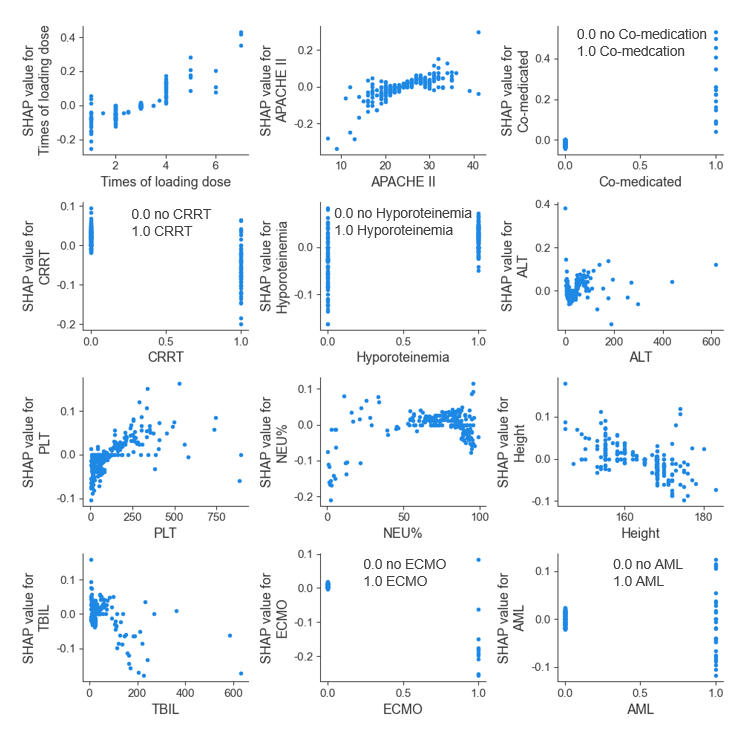


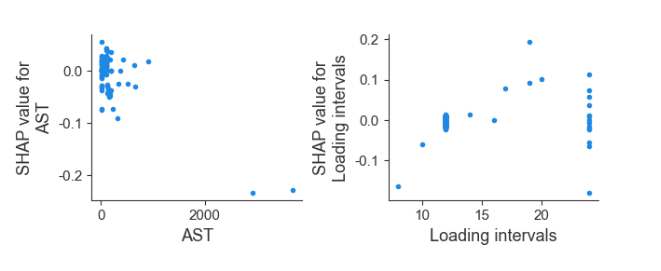


# Figure S1. SHAP dependence plot of model.

Abbreviations: APACHE II, acute physiology and chronic health evaluation II; AST, aspartate aminotransferase; ALT, alanine aminotransferase; TBIL, total bilirubin; NEU%, the percentage of neutrophils; PLT, platelet count; ECMO, extracorporeal membrane oxygenation; CRRT, continuous renal replacement therapy, AML, acute myeloid leukemia.

The SHAP dependence plot (importance ranking 13^th^ to 26^th^) showed how the relevant variable affected the output of the ensemble prediction model. SHAP values for specific relevant variable exceed 0, representing an increased teicoplanin trough concentration.
